# Restless legs syndrome and its association with lower extremity adipose tissue thickness: a novel perspective

**DOI:** 10.1055/s-0045-1811231

**Published:** 2025-08-31

**Authors:** Elif Sarica Darol, Dilcan Kotan

**Affiliations:** 1 Sakarya University, Training and Research Hospital, Neurology Clinic, Sakarya, Türkiye.; 2 Sakarya University, Faculty of Medicine, Department of Neurology, Sakarya, Türkiye.

**Keywords:** Restless Legs Syndrome, Obesity, Subcutaneous Fat

## Abstract

**Background:**

Restless legs syndrome (RLS) is a neurological disorder characterized by unpleasant sensations in the legs often linked to risk factors such as female gender and obesity.

**Objective:**

To explore the correlation between lower extremity subcutaneous adipose tissue thickness (ATT) and RLS, regardless of body mass index (BMI).

**Methods:**

A total of 212 RLS patients and 92 controls were included in this study. The ATT measurements were obtained using a caliper at the midcalf and knee regions and compared between groups. Data analysis was conducted using the IBM SPSS Statistics For Windows (IBM Corp.) software, version 23.0, with multiple logistic regression modeling employed. Values of *p* < 0.05 were considered statistically significant.

**Results:**

The BMI and ATT were significantly higher in female patients ( *p* < 0.001). Notably, patients experienced substantial weight gain in the second decade of symptom onset. Midcalf ATT was significantly lower in RLS patients than controls ( *p* < 0.0005). The likelihood of RLS increased 1.107 times with each unit decrease in midcalf ATT, 1.051 times per increase in year of age, 1.015 times per unit decrease in ferritin level, and 1.072 times per unit decrease in the vitamin D level.

**Conclusion:**

The present study suggests a potential role of leg subcutaneous adipose tissue in RLS pathophysiology, regardless of BMI; it may influence RLS symptoms, offering a novel perspective on its etiology. Further research is warranted to validate these observations and investigate the underlying mechanisms.

## INTRODUCTION

Restless legs syndrome (RLS) is a sensorimotor neurological disorder characterized by unpleasant sensations in the legs, particularly during nighttime and periods of rest. Its prevalence ranges from 5 to 8% among the general population, especially adults. A recent meta-analysis estimates that the prevalence of RLS increases with age, and it negatively affects the quality of daily life, mood, and sleep. 1

Patients commonly describe these sensations as itching, squeezing, tension, jumping, burning, cramping, pain, pulling, or throbbing. 2 These symptoms typically lead to an irresistible urge to move. The most important diagnostic criteria are onset or increase of complaints in inactive situations, such as sitting or lying down and the partial or complete resolution of symptoms with walking or stretching the legs (improvement during activity). 3

Depending on its association with other conditions, RLS is classified as either primary (idiopathic) or secondary (symptomatic). The primary syndrome is often linked to genetic predisposition, whereas the secondary can be associated with various diseases, including diabetes, chronic kidney disease, Parkinson's disease, multiple sclerosis, migraine, pregnancy, and chronic obstructive sleep apnea. 4 Additionally, iron deficiency anemia, female gender, and obesity are recognized as risk factors.

Several studies have explored the link between obesity and RLS, consistently reporting a higher body mass index (BMI) compared with controls. 5 Furthermore, its prevalence is higher among obese individuals. 6 One of the largest population-based studies evaluating the relationship between RLS and obesity emphasized the need to focus specifically on abdominal fat and reported that general obesity and abdominal fat may increase risk of the syndrome. 7

Adipose tissue is not merely an energy storage organ but also metabolically active, functioning as an endocrine organ. In recent years, studies have focused on the type of fat tissue (subcutaneous, visceral, intramuscular) and the areas of fat distribution in the body (such as abdominal) rather than obesity. Their role in the prevention of metabolic diseases and new therapeutic approaches is being investigated. 8 9

Although obesity is considered a risk factor for RLS, it is not yet known whether the main role is played by the metabolic syndromes that accompany it or by its effect on central dopamine receptors. A study found a negative correlation between BMI and the availability of dopamine receptor D2 in obese individuals. 10 Interestingly, exposure to cold temperatures and exercise have been shown to promote the conversion of white adipose tissue into beige, enhancing energy metabolism. 11 Since exercise is one of the most effective ways to alleviate RLS symptoms, and cold applications also provide some relief, the role of adipose tissue in its pathophysiology warrants further investigation.

While most existing studies use BMI to assess overall obesity and body adiposity, the correlation between leg adiposity and RLS in individuals with normal BMI has yet to be investigated.

Given that RLS symptoms improve with movement and cold exposure, we hypothesize that leg adiposity—particularly subcutaneous fat thickness—may play a role in the disease's etiology. This study aims to investigate whether estimated lower extremity adipose tissue thickness (ATT), measured via skinfold thickness, is associated with RLS, independent of BMI. By exploring this novel perspective, we aim to contribute to a deeper understanding of this syndrome's pathophysiology and, potentially, uncover new targets for therapeutic intervention.

## METHODS

Our research protocol was conducted by the principles of the Declaration of Helsinki and was approved by the Ethics in Research Committee of Sakarya University in July 2021, under the number E-71522473–050.01.04–47625–402. All participants provided written informed consent. The current article was previously posted on the *Heliyon* preprint server in June 2024.

### Study design and data collection

This prospective case-control study consists of 212 RLS patients and 92 control cases who applied to the neurology clinic between June 2022 and December 2023. The patient and control groups were informed about the study, and their consent was obtained. Patients diagnosed with primary RLS according to the 2014 International Restless Legs Syndrome Study Group (IRLSSG) diagnostic criteria were included in the study. 3

Sociodemographics, age at RLS symptom onset, lifestyle choices (smoking, consumption of alcohol), and current medical therapy (no medication, vitamin B12 and D, iron supplementation, dopamine agonist, α-2delta ligands, and combined therapy) were recorded. The serum levels of iron (Fe), magnesium (Mg), ferritin, albumin, and of vitamins B12 and D, as well as height and weight, were recorded, and the BMI was calculated for both groups. The laboratory results at the participants' initial visit were included in the study. Blood samples for laboratory analysis were collected after an overnight fast of at least 8 hours.

Depending on the severity of symptoms, patients were divided into four subgroups using the IRLSSG rating scale. 12

### Exclusion criteria

Patients with RLS due to secondary causes, such as diabetes mellitus, kidney diseases, liver diseases, severe malnutrition, deep anemia, antidepressant medication etc. Also, patients with any detected infections, and those taking lithium and calcium channel blockers, were not included.

### Measuring skinfold thickness

Among the methods that evaluate body adiposity and obesity, skinfold thickness measurement is the one used here. 13 Subcutaneous ATT was measured for the first time with the method used by Durnin and Rahaman. 14 It is an easy and cheap method performed with calipers, mostly used in monitoring nutrition and malnutrition.

Skinfold thickness was measured at the gastrocnemius muscles (midcalf) and knees using baseline skinfold calipers 50mm (Fabrication Enterprises Inc.). Participants stood and were instructed to relax their right legs during all measurements, as the right lower extremity is typically preferred for measurement according to the International Society for the Advancement of Kinathropometry (ISAK) protocols. 15 Standardized skinfold measurement techniques were used for the lower extremities' skinfold measurement sites. 16

The same right-handed researcher took measurements within 1 mm of each site by grasping the skin of the participant between the thumb and the forefinger and placing the skinfold caliper approximately 1 cm from the thumb and forefinger.

### Statistical analysis

Descriptive analyses were performed to provide information on the general characteristics of the study population. Shapiro-Wilk's test was used to evaluate whether the distribution of numerical variables was normal. Subsequently, either the Mann-Whitney U test or two independent sample *t* -tests were used to compare the numeric variables between the groups.

Either one-way analysis of variance (ANOVA) or the Kruskal-Wallis *H* test was used to compare the numeric variables among more than two groups. If significant differences were found in comparisons involving more than two groups, pairwise comparisons were conducted using the Sheffe, Tamhane T2, or Dunn tests.

The numeric variables were expressed as mean ± standard deviation (SD) values. A multiple logistic regression model was implemented to determine the ATT and other covariates such as gender, age, BMI, and age at disease onset, among others. The Chi-squared test was used to compare the categorical variables between groups, and the categorical variables were expressed as frequencies and percentages. Valus of *p* < 0.05 were considered statistically significant. Analyses were performed using the IBM SPSS Statistics for Windows (IBM Corp.) software, version 23.0.

## RESULTS

In our study population, the gender distribution was similar between the healthy and RLS groups. There were 22 men and 70 women (76.1%) in the healthy group and 51 men and 161 women (75.9%) in the RLS group ( *p* = 0.979). The baseline characteristics of study participants are shown in Table 1 .

**Table 1:** Baseline characteristics of the study participants

Variable		*n* ( *N* = 212)	%
**Demographics**	Mean age (years)	55.01 ± 14.22	
	Female gender	161	75.9
	Male gender	51	24.1
** Body mass index (kg/m ^2^ ) **	<25 (normal)	5	16.5
	25–30 (overweight)	91	42.9
	≥30 (obese)	86	40.6
**Smoking**	Current	24	11.3
	Never	188	88.6
**Alcohol consumption**	Current	2	0.9
	Never	210	99.0
**Current medical treatment**	No medication	8	3.7
	Vitamin B12 supplementation	18	8.4
	Vitamin D supplementation	11	5.1
	Oral iron supplementation	23	10.8
	Dopamine agonist	51	24.0
	Gabapentin/Pregabalin	9	0.4
	Combined therapy	92	43.3
**Age at disease onset (years)**	≤ 10	160	75.4
	11–20	40	18.8
	≥ 21	12	5.6

Regarding the mean age, the RLS patients were older than the healthy individuals ( *p* < 0.001). The mean BMI distribution between the groups ( *p* = 0.143) was similar, but in the RLS population, 35 cases (16.5%) were of normal weight, 91 (42.9%) were overweight, and 86 cases (40.6%) were obese ( *p* < 0.014), as shown in Table 2 . The significant weight gain seen in patients, especially in the second decade of the disease, and the accompanying increase in BMI and leg ATT, are noteworthy. There were no parameters that correlated with disease severity.

**Table 2:** Comparison of the characteristics of the study sample

		Groups		*p* -value	Effect size
		Control ( *N* = 92): *n* (%)	RLS ( *N* = 212): *n* (%)		
**Gender (female)**		70 (76.1)	161 (75.9)	0.979	0.002
** BMI (kg/m ^2^ ) **	**< 25 (normal)**	29 (31.5)	35 (16.5%)	**0.014**	0.172
	**25–30 (overweight)**	35 (38.0)	91 (42.9%)		
	**≥ 30 (obese)**	28 (30.4)	86 (40.6%)		
**Mean age (years)**		49.24 ± 13.39	55.01 ± 14.22	**0.001**	0.413
**Mean knee ATT (mm)**		16.96 ± 6.35	16.21 ± 7.04	0.446 *	0.109
**Mean midcalf ATT (mm)**		24.93 ± 7.28	22.9 ± 8.59	0.056 *	0.248
**Mean serum Hbg (g/dL)**		13.44 ± 1.53	13.05 ± 1.65	0.058	0.237
**Mean Htc (%)**		40.16 ± 3.99	39.5 ± 4.4	0.218	0.154
**Mean serum ferritin (ng/dL)**		41.41 ± 47.3	29.69 ± 32.86	0.058 *	0.310
**Mean serum iron (µg/dL)**		84.79 ± 52.68	68.74 ± 36.52	0.005*	0.377
**Mean serum vitamin B12 (pg/dL)**		356.48 ± 146.04	420.2 ± 240.76	0.052 *	0.296
**Mean serum vitamin D (pg/mL)**		19.57 ± 9.07	15.08 ± 8.18	< 0.001 *	0.532
**Mean serum magnesium (mg/dL)**		2.25 ± 2.02	2.08 ± 1.71	0.001*	0.095
**Mean serum albumin (g/dl)**		44.08 ± 2.65	42.6 ± 2.99	**0.002**	0.511
** Mean BMI (kg/m ^2^ ) **		27.58 ± 5.05	28.4 ± 4.18	0.143	0.183
**Mean score on the IRLSSG scale**		–	24.88 ± 6.18	–	–

Abbreviations: ATT, adipose tissue thickness; BMI, body mass index; Hbg, hemoglobin; Htc, hematocrit; IRLSSG, International Restless Legs Syndrome Study Group; RLS, restless legs syndrome.

Notes: *The *p* -values were calculated for the Mann-Whitney U test.

The study group's mean age at disease onset was of 8.56 ± 7.41 years, and the mean disease severity score (according to the IRLSSG scale) was of 24.88 ± 6.18. Although significant decreases were detected in serum Fe, vitamin D, magnesium (Mg), and albumin values in the patient population compared with healthy ones ( *p* = 0.005, < 0.001, < 0.001, and = 0.002 respectively). Furthermore, no substantial difference was observed in Ferritin and vitamin B12 values ( *p* = 0.058 and 0.052 respectively), as shown in Table 2 .

**Figure 1:**
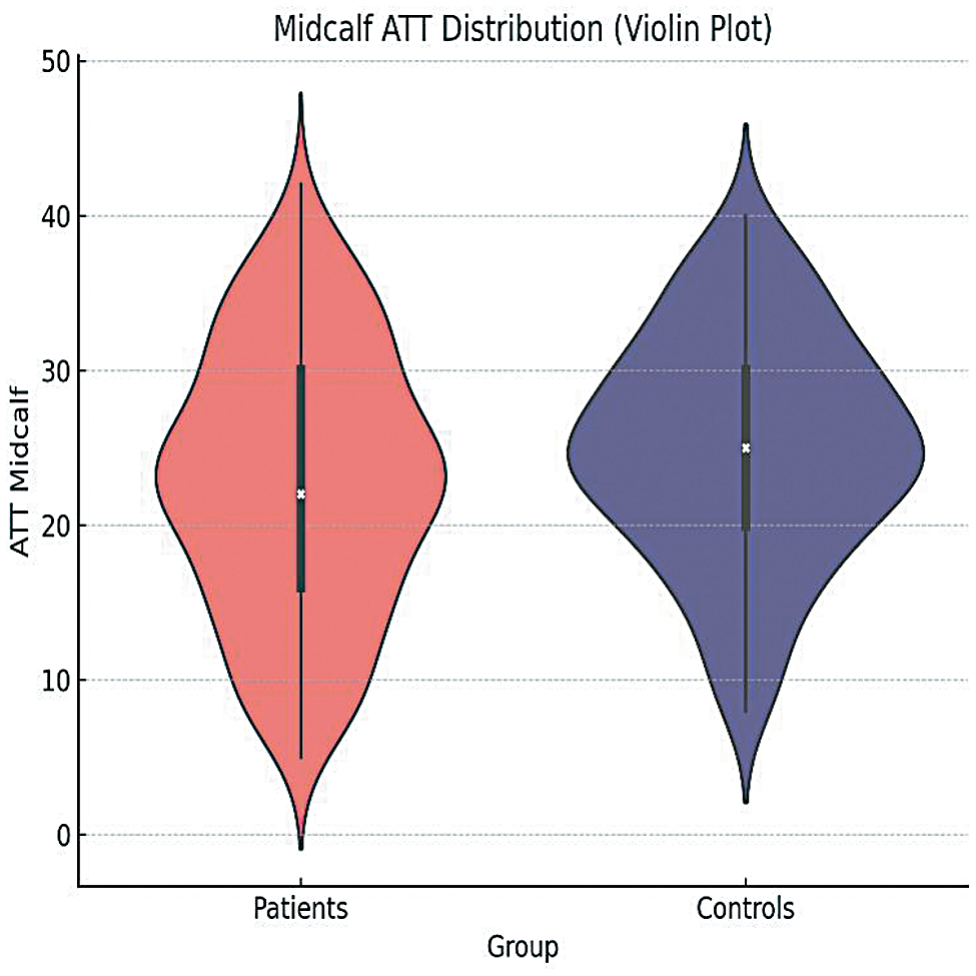
Distribution of midcalf ATT between patients and controls with violin plot. Abbreviation: ATT, adipose tissue thickness. Note: patients: 15.0, controls: 22.0 ( *p* = 0.00005).

**Figure 2:**
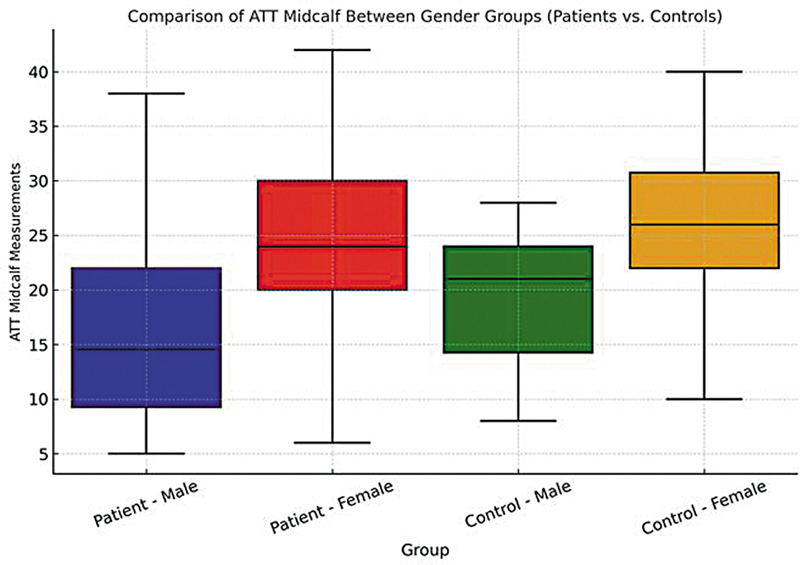
The comparison of the median midcalf ATT values. Abbreviations: ATT, adipose tissue thickness; BMI, body mass index. Notes: male patients: 4.5; female patients: 24.0; male controls: 21.0; female controls: 26.0; with normal BMI.

**Figure 3:**
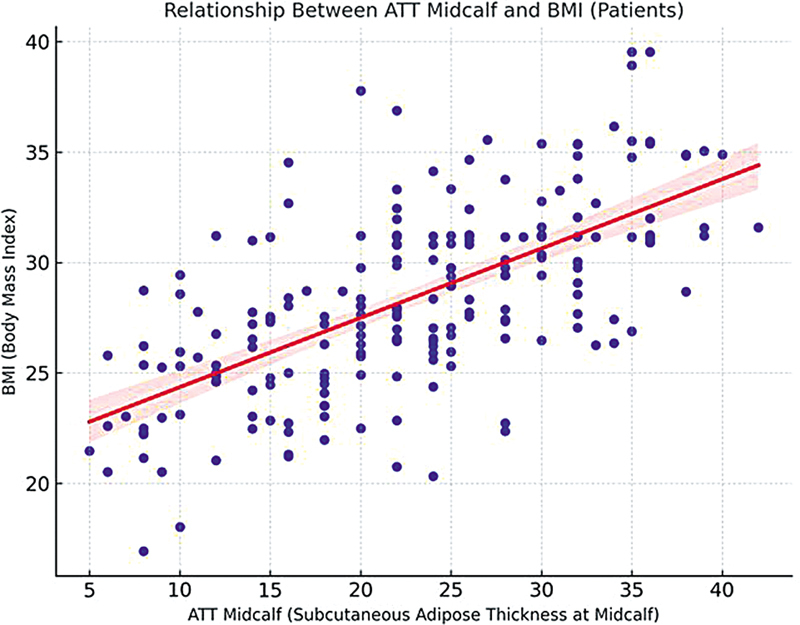
Distribution of positive linear correlation between BMI and midcalf ATT in patients. Abbreviations: ATT, adipose tissue thickness; BMI, body mass index; RLS, restless legs syndrome.

There was no significant difference in knee ATT ( *p* = 0.44) but in midcalf it is almost significantly lower in RLS patients ( *p* = 0.00005). The median midcalf ATT of patients was 15.0 and of the controls was 22.0 ( Figure 1 ). While the lowest average value was found in male patients, for female patients it was lower in the RLS than in the control group ( Figure 2 ). Also, it was lower regardless of BMI, with a linear correlation ( Figure 3 ).

**Figure 4:**
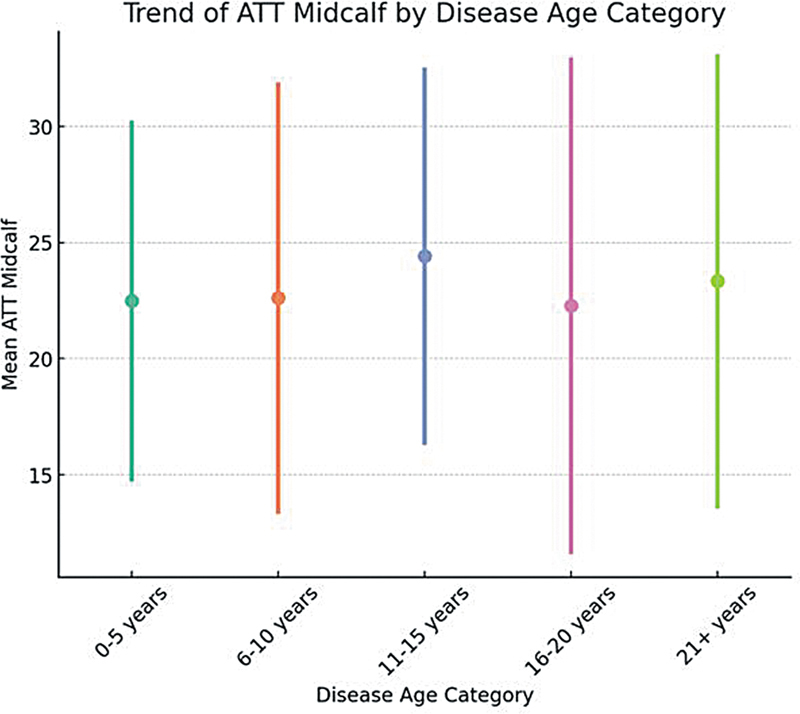
Distribution of median midcalf ATT by disease age. Abbreviation: ATT, adipose tissue thickness. Note: Median ATT value is lower in the first 5 years.

In the RLS group, the mean age distribution of male and female patients and disease duration were similar. The majority of patients were women (75.9%). The BMI, midcalf, and knee subcutaneous ATT were significantly higher in female patients ( *p* < 0.001), while ferritin and Fe values were significantly lower compared with male patients ( *p* < 0.001 and 0.003 respectively), as shown in Table 3 . In the multiple logistic regression model, RLS increases 1.107 (1/0.903) times with 1 unit decrease in the midcalf ATT of patients, 1.051 times with 1-year increase in age, 1.015 (1/0.985) times with 1 unit decrease in ferritin level, and 1.072 times (1/0.933) with 1 unit decrease in vitamin D level ( Table 4 ). In general, the midcalf ATT value tends to increase along with the duration of the disease ( *p* = 0.89). It also shows a lower distribution in those with a short duration of disease, while the distribution is wider in those with medium and long-term disease, while some have higher values ( Figure 4 ).

**Table 3:** Comparison of the characteristics of the male and female RLS patients

		Gender		*p* -value	Effect size
		Male ( *n* = 51)	Female ( *n* = 161)		
**RLS**	**Mean age (years)**	54.96 ± 14.45	55.02 ± 14.20	0.866 *	0.004
	**Mean age at disease onset (years)**	9.71 ± 8.43	8.19 ± 7.05	0.247 *	0.205
	**Mean knee ATT (mm)**	11.76 ± 6.69	17.62 ± 6.57	< 0.001*	0.888
	**Mean midcalf ATT (mm)**	17.1 ± 9.03	24.74 ± 7.59	< 0.001*	0.960
	**Mean serum Hbg (g/dL)**	14.28 ± 1.52	12.66 ± 1.5	< 0.001*	1.076
	**Mean Htc (%)**	42.83 ± 4.12	38.45 ± 3.94	**< 0.001**	1.110
	**Mean serum ferritin (ng/dL)**	56.55 ± 47.4	21.18 ± 20.47	< 0.001*	1.210
	**Mean serum iron (µg/dL)**	82.32 ± 34.46	64.8 ± 36.26	0.003*	0.489
	**Mean serum vitamin B12 (pg/dL)**	444.38 ± 294.31	412.19 ± 220.84	0.618 *	0.134
	**Mean serum vitamin D (pg/mL)**	14.39 ± 5.64	15.25 ± 8.71	0.913 *	0.105
	**Mean serum magnesium (mg/dL)**	1.94 ± 0.13	2.12 ± 1.96	0.707 *	0.104
	**Mean serum albumin (g/dl)**	42.7 ± 4.12	42.57 ± 2.47	0.814	0.046
	**Mean height (cm)**	173.86 ± 8.2	163.63 ± 6.89	< 0.001*	1.417
	**Mean weight (kg)**	80.22 ± 13.47	77.43 ± 10.46	0.489 *	0.247
	** Mean BMI (kg/m ^2^ ) **	26.51 ± 3.9	29 ± 4.1	< 0.001*	0.614
**Mean score on the IRLSSG scale**		23.55 ± 5.7	25.3 ± 6.28	0.101 *	0.285

Abbreviations: ATT, adipose tissue thickness; BMI, body mass index; Hbg, hemoglobin; Htc, hematocrit; IRLSSG, International Restless Legs Syndrome Study Group; RLS, restless legs syndrome.

Notes: *The *p* -values were calculated for the Mann-Whitney U test.

**Table 4:** Multiple logistic regression model to determine the effect of ATT and other covariates on RLS

Dependent variable	Independent variables	β	SE of β	*p* -value	OR	95%CI for OR
**RLS**	**Gender (Female)**	−0.364	0.522	0.486	0.695	0.25–1.934
	**Age**	0.050	0.015	**0.001**	1.051	1.021–1.083
	**Midcalf ATT**	−0.102	0.031	**0.001**	0.903	0.85–0.959
	**Ferritin**	−0.015	0.006	**0.010**	0.985	0.974–0.996
	**Vitamin D**	−0.069	0.021	**0.001**	0.933	0.895–0.973
	** BMI (kg/m ^2^ ) **	0.096	0.056	0.086	1.101	0.87–1.228
	**Constant**	−0.048	1.485	0.974		

Abbreviations: β, regression coefficient; ATT, adipose tissue thickness; BMI, body mass index; OR, odds ratio; RLS, restless legs syndrome; SE, standard error.

## DISCUSSION

The etiopathogenesis of RLS is unknown. While many studies have focused on central nervous system mechanisms such as Fe and dopamine, as well as genetics, there has not been enough research on local etiopathological effects. Female gender and obesity have been identified as risk factors. The hypothesis in our study is to question the role of regional adipose tissue in the lower extremities in the etiopathology of RLS.

The majority of patients in our study group were female, and the average ages of the male and female patients were of 54 and 55 years respectively. This result can be attributed to the inclusion of both newly diagnosed patients and those with chronic RLS in our patient population. Another reason why the average age is over 50 years may be late diagnosis; patients consult a doctor late because they cannot describe their symptoms at the beginning, or doctors do not question RLS patients sufficiently. Moreover, the incidence of the disease increases after the sixth decade of life. 17 18

Our study found a strong correlation between an increase in BMI and knee and midcalf ATT. This supports the claim that there is a correlation between adiposity in the lower extremities and overall body fat. Furthermore, we observed that women had a significantly higher rate of lower extremity adipose tissue than men. It was an expected result that can be attributed to the differences in fat distribution areas between the two genders. 19

However, we detected a significant decrease in midcalf subcutaneous ATT in RLS patients compared with controls. This finding was somewhat unexpected, as obesity has previously been identified as a risk factor for RLS, and one might anticipate that this group would exhibit increased subcutaneous ATT. This finding may be due to changes in the muscle-to-fat ratio in RLS patients, potentially driven by increased motor activity associated with the disorder. Enhanced local muscle activation and circulation in the lower extremities could lead to a reduction in subcutaneous fat stores, resulting in the observed decrease in midcalf ATT.

Low ATT in the lower extremities revealed that metabolic changes in the local fat tissue in the legs should be highlighted, such as low ferritin and vitamin D, which are shown to be metabolic factors for RLS.

Various factors, such as sex hormones and genetic structure, contribute to differences in body adipose tissue distribution among individuals. 20 21 This is the primary lipid storage in the body, being distributed across different regions, including visceral, subcutaneous, and intramuscular areas. In obesity, subcutaneous adipose tissue may not expand adequately to store excess energy, leading to ectopic fat accumulation in other tissues involved in metabolic homeostasis (such as skeletal muscle, liver, and visceral adipose tissues). 22

The accumulation of subcutaneous adipose tissue acts as a physiological storage area for excess energy intake (high-calorie diet) when energy expenditure is limited (physical inactivity). It has been reported that it functions as an endocrine organ and plays a role in the pathophysiology of metabolic syndromes such as diabetes and insulin resistance. 9 Different colors of adipose tissue have been described, and it has been reported that the beige one in particular transforms into brown in response to exercise, exposure to cold, and certain hormones. 23 Clinical studies have shown that brown and beige masses are much higher and more active in women than in men, and strategies to increase activity of the first have been focused on developing individual and gender-based treatment approaches. 24

A very recent survey conducted on RLS patients also reported that the healing effect of exercise was greater in patients with a low BMI. 25 As an anthropometric index, BMI is used to classify adipose mass and obesity. However, the relationship between BMI and the body adipose tissue mass is not strong enough to accurately predict adiposity, as it does not account for body composition (that is, skeletal muscle mass). 22 Using this index alone may prevent accurate and objective scientific results, especially due to the different adipose distribution between genders. Additionally, BMI does not distinguish between lipid structures (subcutaneous, visceral, or intramuscular) of different colors (brown, beige, or white) and functions that make up the adipose tissue, making it difficult to fully assess adiposity.

The RLS affects women more frequently than men, with a reported prevalence of 30 to 50% rate. 26 While women tend to have more adipose tissue in the legs and hips, men typically accumulate more central tissue. 19

To date, only one study 27 has investigated the relationship between obesity and RLS by measuring subcutaneous adipose tissue, which is similar to our study. In this multicenter, multi-ethnic, prospective cohort study 27 involving 2,704 healthy pregnant women aged 18 to 40 years, without a prior diagnosis of RLS, body composition measurements—including prepregnancy BMI, waist circumference, and subscapular and triceps skinfold thickness—were conducted to assess the risk of developing RLS during pregnancy. The study found no significant association between general adiposity indicators, such as prepregnancy BMI and waist circumference, and the risk of this syndrome. However, a significant positive association was observed between the total subscapular and triceps skinfold thickness measured in early pregnancy (8–13 weeks of gestation) and the subsequent development of RLS. These findings led the authors to conclude that increased subcutaneous adiposity may represent a potential new risk factor for the syndrome's onset in pregnancy, independent of overall adiposity. 27

Although there is a methodological similarity, the referenced study focused solely on pregnant women and, due to edema in the legs of pregnant women, measured only subcutaneous ATT in the upper extremities and back. Furthermore, because weight gain during pregnancy varies both metabolically and regionally under the influence of hormonal changes, we believe that the results of this study cannot be generalized to patients with primer RLS.

It is known that RLS often begins in one leg—sometimes even in a single ankle—and primarily affects areas below the knee before progressing to the other leg and, eventually, the whole body over time. The fact that patients experience relief from symptoms through maneuvers such as walking, movement, or massage—one of the diagnostic criteria for RLS—along with the emergence of symptoms at rest, suggests that physical activity and subcutaneous adiposity deserve further investigation from a pathophysiological perspective.

Many studies highlight the role of vascular changes, blood flow, and oxygenation in RLS pathology indicating metabolic alterations in the vascular and muscle tissue of the legs. 28 29 30 Our study has developed a novel perspective focused specifically on regional subcutaneous adipose tissue in the etiopathogenesis of the disease by demonstrating its reduction in the local midcalf of primary RLS patients.

However, our results should be considered preliminary due to the caliper measurement method. While they are inexpensive and easy to perform, there is a high margin of error, even when conducted by the same individual. Future studies should employ more objective methods, such as magnetic resonance imaging (MRI) or dual-energy X-ray absorptiometry (DEXA) to evaluate the muscle-to-adipose tissue ratio and their relationship with exercise in the legs more accurately. 31 32

Vitamin D and iron metabolism, particularly ferritin and iron, show a strong correlation with RLS, which corroborates previous research. According to our findings, albumin levels are significantly lower in RLS patients. In another study we conducted on this topic, we observed that albumin levels were significantly higher compared with controls. 33 These contradictory results indicate that albumin levels can be influenced by various factors, including diet, infection, and exercise. 34

In 2012, Abate and Chandalia 8 examined the influence of body adipose distribution on metabolic health, and they posited that subcutaneous ATT, particularly when accumulated in the lower body regions, might have a protective effect against metabolic complications. They emphasized that both quantity and distribution of body fat significantly impact metabolic risks. They concluded that the distribution of adipose tissue should be considered in obesity treatment and the prevention of metabolic diseases. 8 Drawing from this, we suggest that future research should focus more on the role of local subcutaneous adipose tissue in the pathophysiology of RLS instead of general body adiposity.

## Limitations

One limitation of our study is that our patient population was not limited to recently diagnosed primary RLS patients, including those who were already being treated in the chronic period. Newly-diagnosed and chronic RLS patients should be analyzed separately.

The most significant limitation of our study is that we measured ATT using a caliper, which is prone to errors. There is a high risk of incorrect measurement with this tool, depending on the professionals' experience. 35 However, caliper measurements for ATT are a direct and quick method for regional adiposity.

Since the study is cross-sectional and only examines data from a certain period, it can only show correlations rather than determine the factors that lead to RLS. Longitudinal studies should be conducted to investigate how adipose tissue changes affect the development of RLS over time. Although the study showed a relationship between ATT loss and RLS, new studies should be planned to observe the effect of regional adiposity on RLS symptoms to determine whether this condition causes RLS.

In conclusion, the role of subcutaneous adipose tissue in the lower extremities of patients with chronic RLS warrants further investigation. Our study suggests a possible influence of leg subcutaneous adipose tissue, regardless of BMI, and proposes the potential role of the muscle-adipose tissue ratio in metabolism and therapeutic strategies. To gain a deeper understanding of the relationship between subcutaneous adipose tissue and the onset of RLS, longitudinal and large-scale studies are needed.
